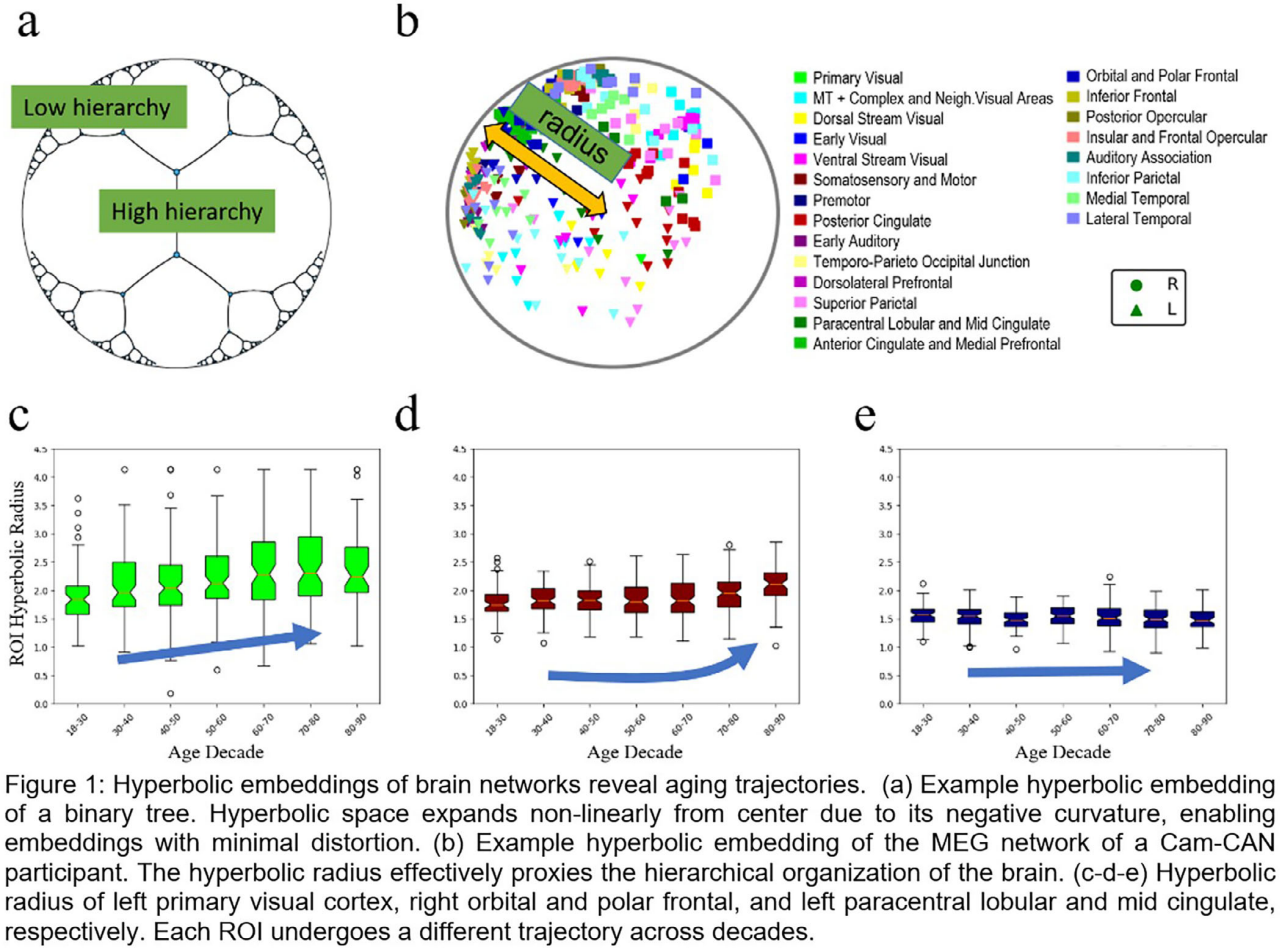# Determining aging trajectories though hyperbolic embeddings of MEG brain networks

**DOI:** 10.1002/alz.086649

**Published:** 2025-01-09

**Authors:** Hugo Ramirez, Davide Tabarelli, Arianna Brancaccio, Paolo Belardinelli, Elisabeth B Marsh, Michael E Funke, John C Mosher, Fernando Maestú, Mengjia Xu, Dimitrios Pantazis

**Affiliations:** ^1^ Massachusetts Institute of Technology, Cambridge, MA USA; ^2^ University of Trento, Trento, Autonomous Province of Trento Italy; ^3^ Johns Hopkins University, Baltimore, MD USA; ^4^ University of Texas Health Science Center at Houston, Houston, TX USA; ^5^ Complutense University of Madrid, Madrid, Madrid Spain; ^6^ Massachusetts Institute of Technology, Cambidge, MA USA; ^7^ New Jersey Institute of Technology, New Jersey, NJ USA

## Abstract

**Background:**

Investigating age‐related changes in MEG brain networks offers significant potential for comprehending aging trajectories and unveiling anomalous patterns associated with neurodegenerative disorders, such as Alzheimer's disease. In this study, we extended a deep learning model called Fully Hyperbolic Neural Network (FHNN) to embed MEG brain connectivity graphs into a Lorentz Hyperboloid model for hyperbolic space. Through these embeddings, we then explored the impact of aging on brain functional connectivity across multiple decades.

**Method:**

We analyzed data from 587 participants enrolled in the Cambridge Centre for Ageing and Neuroscience (Cam‐CAN) longitudinal study. Notably, we introduced a unique metric—the radius of the node embeddings—which effectively proxies the hierarchical organization of the brain. We leveraged this metric to (i) assess whether we can decode age‐related information, and (ii) characterize subtle hierarchical organization changes of various brain subnetworks attributed to the aging process.

**Result:**

Our decoding results revealed that the hyperbolic radius carries substantially more age‐related information compared to all other conventional graph‐theoretic measures examined, underscoring the effectiveness of employing hyperbolic embeddings to characterize the aging process. An examination of hyperbolic radius alteration patterns across decades exposed numerous subnetworks showcasing a decline in hierarchy during aging, with some displaying gradual changes and others undergoing rapid transformations in the aging brain (illustrated in Figure 1cde).

**Conclusion:**

Overall, our study presented the first evaluation of hyperbolic embeddings in MEG brain networks, introduced a novel measure of brain hierarchy, and used this measure to highlight aging trajectories in the large cohort of the Cam‐CAN dataset. A prominent finding was the reduction of hierarchy across a substantial number of subnetworks throughout the aging brain. This hierarchy reduction could imply a shift in the brain network configuration impairing cognitive processes.